# The Different Roles of MET in the Development and Treatment of Cancer

**DOI:** 10.3390/cancers15205087

**Published:** 2023-10-21

**Authors:** Jens Mollerup, Jan Trøst Jørgensen

**Affiliations:** 1 Pathology Division, Agilent Technologies Denmark ApS, Produktionsvej 42, 2600 Glostrup, Denmark; 2 Department: Medical Sciences, Dx-Rx Institute, Baunevaenget 76, 3480 Fredensborg, Denmark; jan.trost@dx-rx.dk

This Special Issue features contributions from leading international researchers in the field of MET (hepatocyte growth factor (HGF) receptor) biology and therapeutics. Recent discoveries regarding non-small cell lung cancer (NSCLC) and gastric cancer, as well as advancements in the detection of *MET* aberrations and new targeted therapies for MET-driven cancers and resistance mechanisms are explored. Aberrations in the *MET* gene leading to impaired MET-dependent signaling have been identified as primary and secondary drivers of cancer development. To optimally detect and potentially counteract these effects with mono- or combination therapy, it is crucial to understand the underlying cellular mechanisms involved in MET-dependent cancer cell development and growth.

Dysregulated MET signaling, which predisposes cells to cancer development, can occur due to MET overexpression, *MET* gene amplification, MET kinase mutations, mutations resulting in *MET* exon 14 skipping, *MET* rearrangements, and *MET* fusions [1]. A variety of technologies are used to detect aberrations linked to MET in clinical samples, including immunohistochemistry (IHC), next-generation sequencing (NGS) of DNA or RNA, Sanger sequencing of RNA, reverse transcription–polymerase chain reaction (RT-PCR), digital droplet PCR (ddPCR), nanostring nCounter, in situ hybridization (ISH), and mass spectrometry [1,2,3,4,5].

Das et al. [3] revealed two novel noncanonical *MET* splice variants leading to *MET* exon 14 skipping in NSCLC. Their study highlights the importance of recognizing noncanonical splice events by integrating next-generation sequencing data with in silico predictions in order to assess the potential impact of mutations. Additionally, they demonstrated the potential of routinely using cytology slides for RNA-based NGS testing.

Feldt et al. [6] report that the progression of NSCLC following treatment with epidermal growth factor receptor (EGFR) tyrosine kinase inhibitor (TKI) frequently involves changes in the *MET* gene, including *MET* amplification. In NSCLC patients who had progressed on osimertinib, early clinical trials have shown promising antitumor activity following a combination therapy with the third-generation EGFR TKI lazertinib and the MET-EGFR bispecific antibody amivantamab.

Gamerith et al. [7] utilized ISH and NGS to examine genetic alterations in lung cancer patients exposed to radon. Their study unexpectedly revealed a higher frequency of *MET* alterations in radon-exposed patients compared with the control group.

In a systematic review following PRISMA guidelines, Bodén et al. [8] examined 22 published papers relating to clinical trials on MET, lung cancer, and targeted MET therapies from the Embase and PubMed databases between 2013 and February 2023. Six clinical trials indicated favorable outcomes of MET inhibitor treatment in terms of progression-free survival (PFS) and the overall response rate (ORR), while two clinical trials failed to show a beneficial effect of adjunctive MET-targeted therapy.

*MET* amplification is known as a pivotal biomarker, but establishing the optimal thresholds for recognizing *MET* amplification in patient samples is challenging [4,9]. The determination of the *MET* copy number can be achieved through ISH and NGS, and according to Kumaki et al. [4], a significant challenge is the distinction between focal amplification and polysomy. As ISH exhibits higher sensitivity compared with NGS, ISH is considered the gold standard for copy number determinations and detection of *MET* amplification [5]. With regard to metastatic NSCLC, Qin et al. [10] elaborated on how *MET* amplification plays a key role in resistance to tyrosine kinase inhibitors (TKI) and discuss strategies to overcome this.

By employing a proteomics approach, Jie et al. [2] presented evidence that the four plasma biomarkers MYH9, GNB1, ALOX12B, and HSD17B4 could substitute or complement response prediction by using fluorescence ISH (FISH) or IHC in patients receiving MET inhibitors.

To gain further insights into the mechanisms underlying drug resistance, Cecchi et al. [11] investigated the path to rilotumumab resistance in a glioblastoma cell line that was dependent on autocrine signaling via HGF and MET. Rilotumumab is an investigational, fully human monoclonal antibody that binds HGF and prevents HGF-mediated activation of MET. Resistance towards rilotumumab was found to depend on *MET* and *HGF* amplification, excessive production and misfolding of HGF, induction of endoplasmic reticulum stress-response signaling, and an increased uptake and degradation of rilotumumab. Collectively, these mechanisms enable resistant glioblastoma cells to sustain adequate HGF-dependent MET signaling, thereby promoting survival and cell growth.

In a narrative review by Hsu et al. [12], the development of capmatinib from preclinical studies to its approval for treatment of MET-driven NSCLC was presented. Capmatinib received FDA approval in 2022 for advanced non-small cell lung cancer (NSCLC) with *MET* exon 14 skipping mutations. Hsu et al. specify that ongoing clinical research aims to improve the treatment efficacy and explore new indications for capmatinib, including addressing *MET* amplification that has developed following EGFR TKI resistance. Combination therapies with capmatinib and other agents are also under investigation. Based on data from various clinical trials, Hsu et al. compared the efficacy outcomes of the three approved MET TKIs—capmatinib, tepotinib, and savolitinib—in the treatment of patients with metastatic NSCLC with *MET* exon 14 skipping mutations [12]. These clinical trials demonstrated an ORR range of 41% to 68%, dependent on the patient type and previous treatment history, and a corresponding PFS range of 6.8 to 12.4 months.

Similarly, the review by Zhu et al. [13] examined the development of the highly selective MET-TKI, savolitinib. Savolitinib obtained conditional approval in China in 2021 for the treatment of NSCLC with *MET* exon 14 skipping mutations, and this review outlines preclinical models, phase I studies in Chinese patients, and the TATTON study combining savolitinib with osimertinib. The authors conclude that both preclinical and clinical evidence support the efficacy and tolerability of savolitinib in treating advanced NSCLC patients with *MET* exon 14 skipping mutations. Furthermore, when using savolitinib in conjunction with EGFR-TKIs, the authors indicate that it demonstrates potential in terms of overcoming treatment resistance stemming from both *MET* amplification and MET overexpression.

The review by Van Herpe and Van Cutsem [9] focuses on the role of MET in gastric cancer and discusses the clinical significance of MET-targeted therapies. The review also explores various diagnostic assays, such as immunohistochemistry, FISH, H-score, and NGS. The authors highlight the challenges of identifying patients who will benefit from treatment with MET inhibitors due to the variability in diagnostic assays. They note that the success of MET-targeted therapy in gastric cancer appears to be limited, with consistent limitations such as the number of patients, differences in inclusion criteria, and diagnostic assays for patient selection in clinical trials with TKIs. However, the VIKTORY umbrella trial stands out as an exception, where a cohort of gastric cancer patients with *MET* amplification received treatment with savolitinib and achieved an ORR of 50%. The authors emphasize that a major challenge remains in establishing clinically significant cut-off values for *MET* amplification and MET overexpression to guide treatment-related decisions.

The development of companion diagnostic assays for targeted cancer therapies requires a profound understanding of the pathophysiology and the drug’s mechanism of action [5]. In the case of MET inhibitors, unexplored avenues requiring further investigation remain. Over the past decade, intensive research has been carried out to develop MET-targeting drugs, including both small-molecule inhibitors and antibody-based drugs. As described in this Special Issue of *Cancers*, only a few MET inhibitors have obtained regulatory approval, and this is so far limited to the treatment of metastatic NSCLC patients. A key challenge in the development of MET inhibitors appears to be related to identifying the appropriate predictive biomarker to guide drug use. In NSCLC, a small number of MET TKIs have demonstrated efficacy when patients are selected based on *MET* exon 14 skipping mutations. Another potential predictive biomarker for MET-targeted therapy is *MET* amplification, identified as a resistance mechanism in patients with EGFR-mutated NSCLC. However, their full potential in both NSCLC and gastric cancer remains to be fully explored, which we hope to witness in the coming years.
